# Thymosin beta 4 is associated with RUNX2 expression through the Smad and Akt signaling pathways in mouse dental epithelial cells

**DOI:** 10.3892/ijmm.2015.2118

**Published:** 2015-03-02

**Authors:** HIROTAKA SOMEYA, HIROAKI FUJIWARA, KENGO NAGATA, HIROKO WADA, KANA HASEGAWA, YURIE MIKAMI, AKIKO JINNO, HIDETAKA SAKAI, KIYOSHI KOYANO, TAMOTSU KIYOSHIMA

**Affiliations:** 1 Laboratory of Oral Pathology, Division of Maxillofacial Diagnostic and Surgical Sciences, Kyushu University, Fukuoka 812-8582, Japan; 2 Section of Implant and Rehabilitative Dentistry, Kyushu University, Fukuoka 812-8582, Japan; 3 Department of Endodontology and Operative Dentistry, Division of Oral Rehabilitation, Kyushu University, Fukuoka 812-8582, Japan; 4 Section of Oral and Maxillofacial Oncology, Division of Maxillofacial Diagnostic and Surgical Sciences, Faculty of Dental Science, Kyushu University, Fukuoka 812-8582, Japan

**Keywords:** thymosin beta 4, RUNX2, calcification, odontogenesis, knockdown assay, Smad, Akt

## Abstract

In previous studies by our group, we reported that thymosin beta 4 (Tb4) is closely associated with the initiation and development of the tooth germ, and can induce the expression of runt-related transcription factor 2 (RUNX2) during the development of the tooth germ. RUNX2 regulates the expression of odontogenesis-related genes, such as amelogenin, X-linked (Amelx), ameloblastin (Ambn) and enamelin (Enam), as well as the differentiation of osteoblasts during bone formation. However, the mechanisms through which Tb4 induces the expression of RUNX2 remain unknown. In the present study, we employed a mouse dental epithelial cell line, mDE6, with the aim to elucidate these mechanisms. The mDE6 cells expressed odontogenesis-related genes, such as Runx2, Amelx, Ambn and Enam, and formed calcified matrices upon the induction of calcification, thus showing characteristics of odontogenic epithelial cells. The expression of odontogenesis-related genes, and the calcification of the mDE6 cells were reduced by the inhibition of phosphorylated Smad1/5 (p-Smad1/5) and phosphorylated Akt (p-Akt) proteins. Furthermore, we used siRNA against Tb4 to determine whether RUNX2 expression and calcification are associated with Tb4 expression in the mDE6 cells. The protein expression of p-Smad1/5 and p-Akt in the mDE6 cells was reduced by treatment with Tb4-siRNA. These results suggest that Tb4 is associated with RUNX2 expression through the Smad and PI3K-Akt signaling pathways, and with calcification through RUNX2 expression in the mDE6 cells. This study provides putative information concerning the signaling pathway through which Tb4 induces RUNX2 expression, which may help to understand the regulation of tooth development and tooth regeneration.

## Introduction

Mammalian tooth development is regulated by signaling cascades involving various genes (1–5). In our previous studies, (6–19) we reported the genes that were differentially expressed between mouse mandibles on embryonic day (E)10.5 and E12.0 using a cDNA subtraction method (6), and that these genes are associated with tooth development. Thymosin beta 4, X-linked (Tb4) was one of the genes highly expressed in the E12.0 mandible (6). Tb4 is closely associated with the differentiation of dental epithelial cells during tooth development (8,17). Tb4-overexpressing transgenic mice were observed to have enamel hypoplasia-like abnormal tooth development (20). These results suggest that Tb4 plays an important role in tooth development.

Tb4 consists of 43 amino acid residues, and is a 4.9-kDa actin-sequestering peptide (22). Tb4 plays a role in cell motility by regulating the polymerization and depolymerization of actin (23). We have previously demonstrated that Tb4 is tightly associated with tooth morphogenesis through runt-related transcription factor 2 (Runx2) expression in the organ-cultured tooth germ following Tb4 knockdown (17). The expression of odontogenesis-related genes, such as Runx2, amelogenin, X-linked (Amelx), ameloblastin (Ambn) and enamelin (Enam) was induced in non-odontogenic human keratinocytes transfected with a Tb4 expression vector (19). Tb4 may participate in tooth development through the regulation of Runx2 expression. In addition, Smart *et al* (24) previously reported that the mouse epicardium pre-treated with Tb4 was induced to re-express Wt1, a key embryonic epicardial gene, and that the tissue was converted into cardiomyocytes. Taken together, these previous findings suggest that Tb4 has the ability to induce gene expression.

RUNX2 is a key differentiation marker of osteoblasts and regulates bone formation. The knockdown of type II/III RUNX2 expression has been shown to reduce the calcification of calvarial cells (25). Additionally, RUNX2 is tightly involved in calcification during tooth formation (26–28) and regulates the expression of odontogenesis-related genes (9,17,19,29–31). RUNX2 expression is observed at various stages in tooth development (32,33). Therefore, RUNX2 is considered to play an important role in the development and calcification of the tooth germ.

Various signaling pathways involving Smad, PI3K-Akt, MAPK, Hedgehog, Wnt/β-catenin and so on have been reported to be upstream of RUNX2 expression during bone formation (34,35). Some of these signaling pathways are also associated with RUNX2 expression during tooth development (21,36,37). Tb4 has been shown to promote MAPK and Smad signaling to induce the formation of calcified materials in human dental pulp cells (21). Tb4 activates the JNK signaling pathway to increase the expression of pro-inflammatory cytokines in cancer cells (38), and induces the upregulation of ERK phosphorylation to increase the resistance of cancer cells to paclitaxel (39). These studies suggest that Tb4 activates signaling pathways upstream of RUNX2. However, little is known about the role of Tb4-RUNX2 signaling in the developing tooth germ.

In the present study, we therefore investigated Tb4-RUNX2 signaling in the mouse dental epithelial cell line, mDE6. Our results demonstrated that the Smad and PI3K-Akt pathways may be involved in tooth development, and provide new information concerning the signaling pathway from Tb4 to RUNX2 expression in the mDE6 cells, which may help to understand the regulation of tooth development and regeneration.

## Materials and methods

### Cell lines and cell culture

The mouse dental epithelial cell line, mDE6, established from mouse tooth germ was kindly provided by Professor Satoshi Fukumoto (Tohoku University, Sendai, Japan). The mDE6 cells were cultured in DMEM/F12 medium supplemented with 10% fetal bovine serum, 100 U/ml penicillin and 100 mg/ml streptomycin (all from Life Technologies, Carlsbad, CA, USA) in a humidified atmosphere of 5% CO_2_ at 37°C, as previously described (17,18).

### Induction of calcification in cell culture

The mDE6 cells were seeded in Ø35 mm dishes and were incubated in culture medium without antibiotics. At 48 h after seeding, the induction of calcification began with the use of calcified induction medium (CIM), which was culture medium containing 50 *μ*g/ml ascorbic acid and 10 mM β-glycerophosphate. The protocol for the induction of calcification was based on that of a previous study (19). The CIM was changed every 3 days. After the induction of calcification for 21 days, some dishes were fixed with 4% paraformaldehyde (PFA) in 0.01 M phosphate-buffered saline (pH 7.2) and stained with 1% Alizarin red S (ALZ) or von Kossa (Kossa) for histological evaluation to identify calcification. The others were analyzed by reverse transcription-quantitatvie polymerase chain reaction (RT-qPCR) or by western blot analysis.

### Semi-quantitative RT-PCR

RT-qPCR was performed as described in previous studies (17,19). In brief, total RNA was isolated from the mDE6 cells using the SV Total RNA Isolation system (Promega, Madison, WI, USA), and was reverse transcribed using the SuperScript^®^ VILO™ cDNA Synthesis kit and master mix (Life Technologies) according to the manufacturer's instructions. The expression of target genes was analyzed using the Thermal Cycler Dice^®^ Real-Time system, with SYBR^®^ Premix Ex Taq™ II (both from Takara, Shiga, Japan). The primers used are listed in Table I. Glyceraldehyde-3-phosphate dehydrogenase (Gapdh) was used as an endogenous reference gene for relative quantifications. The relative expression level of each target gene was normalized using the ΔΔCT comparative method based on the reference gene threshold cycle (CT) values, as previously described (16,17,19).

### Western blot analysis

Sodium dodecyl sulfate-polyacrylamide gel electrophoresis (SDS-PAGE) and western blot analysis were performed as previously described (19). Briefly, each sample of total protein (10 *μ*g/lane) was fractionated by 10 or 15% gel electrophoresis, and the proteins were transferred onto a polyvinylidene difluoride membrane (Bio-Rad, Hercules, CA, USA). The membrane was incubated with the primary antibodies (Table II). Bound antibodies were reacted with a 1:5,000 dilution of HRP-conjugated secondary antibodies, and were visualized using the ECL Prime Western Blotting Detection system (GE Healthcare, Little Chalfont, UK). Emitted light was detected using the ImageQuant LAS 4000 (GE Healthcare), a cooled CCD-camera. In the semi-quantitative analyses of the levels of protein expression, the intensity of the bands was measured using the ImageQuant TL software (GE Healthcare). GAPDH was used as an internal control protein. The ratio of target protein/GAPDH based on the intensity of the bands was calculated, as previously described (17,19). After the detection of a targeted phosphorylated protein, the membrane was reprobed to detect the targeted non-phosphorylated protein on the same membrane.

### Inhibition assays

LDN193189 [an inhibitor of the phosphorylated (p-) Smad1/5/8 pathway] was obtained from AdooQ BioScience (Irvine, CA, USA). Triciribine (a p-Akt pathway inhibitor) and dimethyl sulfoxide (DMSO) were obtained from Wako Chemical Inc. (Osaka, Japan). The mDE6 cells were cultured in DMEM/F12 medium with LDN193189 or triciribine in Ø35 mm dishes for 48 h. The final concentration of DMSO in the medium was 0.1% (v/v). The final concentrations of the inhibitors were: 50 and 500 nM LDN193189 and 250 and 2,500 nM triciribine. The mDE6 cells were also cultured in CIM with LDN193189 or triciribine for 10 days when the mDE6 cells were fully confluent in Ø35 mm dishes. The CIM with the inhibitor was changed every other day. The cells treated with DMSO alone were used as the controls. All samples were analyzed by RT-qPCR, western blot analysis or ALZ staining.

### Transfection with siRNA against Tb4

The cells were seeded in Ø35 mm dishes in culture medium without antibiotics. At 24 h after seeding, the cells were treated with siRNAs according to the manufacturer’s instructions using the Lipofectamine^®^ RNAiMAX Transfection Reagent (Life Technologies). Three siRNAs against Tb4 (siRNA-1, -2 and -3) were designed and prepared. Their target sites were different. siRNA (final concentration 10 nM) was transfected into the cells with the aid of 4 *μ*l of the RNAiMAX reagent. The cells were incubated with the siRNA complex for 48 h. A universal negative control siRNA (Sigma-Aldrich, St. Louis, MO, USA) was used as a negative control. The transfected cells were analyzed by RT-qPCR and western blot analysis.

### Analysis of the effect of Tb4 inhibition using siRNA on the calcification of mDE6 cells

At 48 h after siRNA transfection (as mentioned above), the induction of calcification began with the use of CIM. Transfection of the mDE6 cells with siRNA against Tb4 was repetitively performed using Lipofectamine RNAiMAX every time the CIM was changed. After the induction of calcification for 10 days, the cells were fixed with 4% PFA and stained with ALZ.

### Statistical analysis

All the experiments were independently performed at least in triplicate. All values are presented as the means ± SD. A one-way ANOVA with the Tukey-Kramer comparison test was used to analyze the data obtained with by RT-qPCR and western blot analysis. Differences resulting in P-values of <0.05 or 0.01 were considered to be statistically significant.

## Results

### Calcification of the mDE6 cells

**Figure 1:**
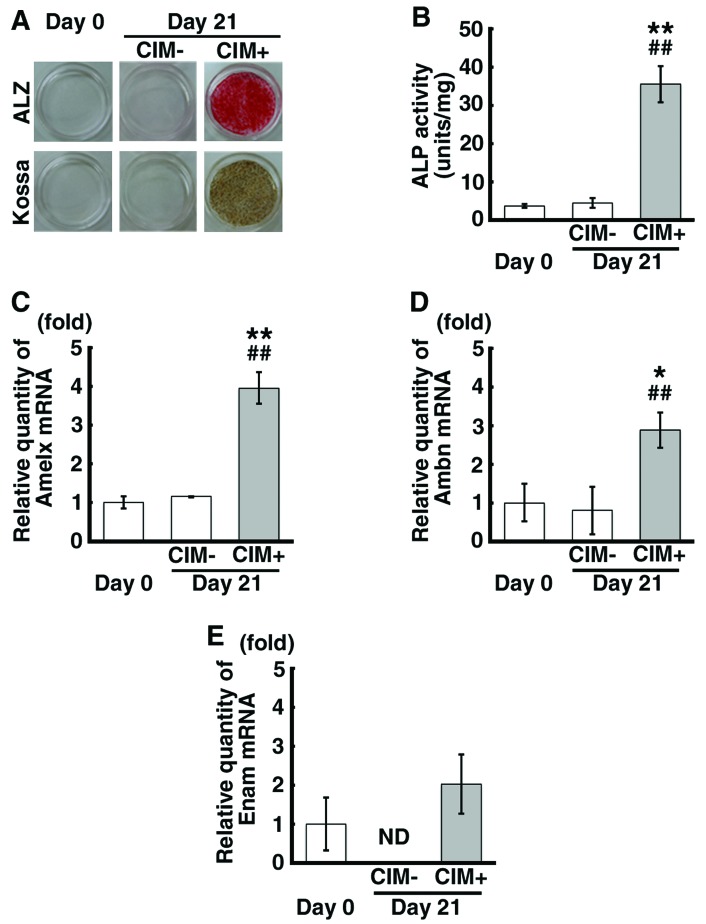
The calcification and expression of odontogenesis-related genes in the mDE6 cells upon the induction of calcification. (A) The mDE6 cells incubated with calcified induction medium (CIM) for 21 days showed the formation and calcification of matrix. The calcified matrices in the mDE6 cells were positive for Alizarin red S (ALZ; upper panel) and von Kossa (Kossa; lower panel) staining. The control without CIM treatment (CIM-) was negative for ALZ and Kossa staining, as were the cells on day 0. (B) The enzymatic activity of alkaline phosphatase (ALP) was significantly increased in the mDE6 cells treated with CIM for 21 days. (C–E) The expression levels of (C) amelogenin, X-linked (Amelx), (D) ameloblastin (Ambn) and (E) enamelin (Enam) were increased in the mDE6 cells treated with CIM compared with those in CIM- cells and the cells on day 0. The data are the means ± SD from triplicate samples. ^*^P<0.05 and ^**^P<0.01 vs. day 0; and ^##^P<0.01 vs. CIM- by a one-way ANOVA with the Tukey-Kramer comparison test. ND, not detectable.

We wished to determine whether the formation of calcified material was induced in mDE6 cells by the use of CIM, as well as whether the activity of alkaline phosphatase (ALP) is altered during calcification and whether the cells express odontogenesis-related genes, such as Amelx, Ambn and Enam. Calcification, as indicated by positive ALZ and Kossa staining, was observed in the mDE6 cells cultured in CIM for 21 days (CIM+ cells) (Fig. 1A), while no calcification was noted in the cells cultured without CIM (CIM-cells). ALP activity was significantly increased in the CIM+ cells (Fig. 1B). The mRNA expression levels of Amelx and Ambn were significantly increased in the CIM+ cells (Fig. 1C and D). Although there were no significant differences observed in Enam mRNA expression between the CIM+ cells and the controls (CIM-cells) and the cells just before the induction of calcification (cells on day 0), the Enam mRNA expression appeared to be increased in the CIM+ cells (Fig. 1E). There were no marked differences observed in the expression levels of these genes in the CIM- cells compared with those observed on day 0 (Fig. 1C–E). These results indicate that the mDE6 cells have the ability to express odontogenesis-related genes and form calcified material, depending on the culture conditions, and partially show the characteristics of odontogenic epithelial cells *in vivo*.

### Signaling pathways upstream of Runx2 expression in the mDE6 cells cultured in CIM

**Figure 2:**
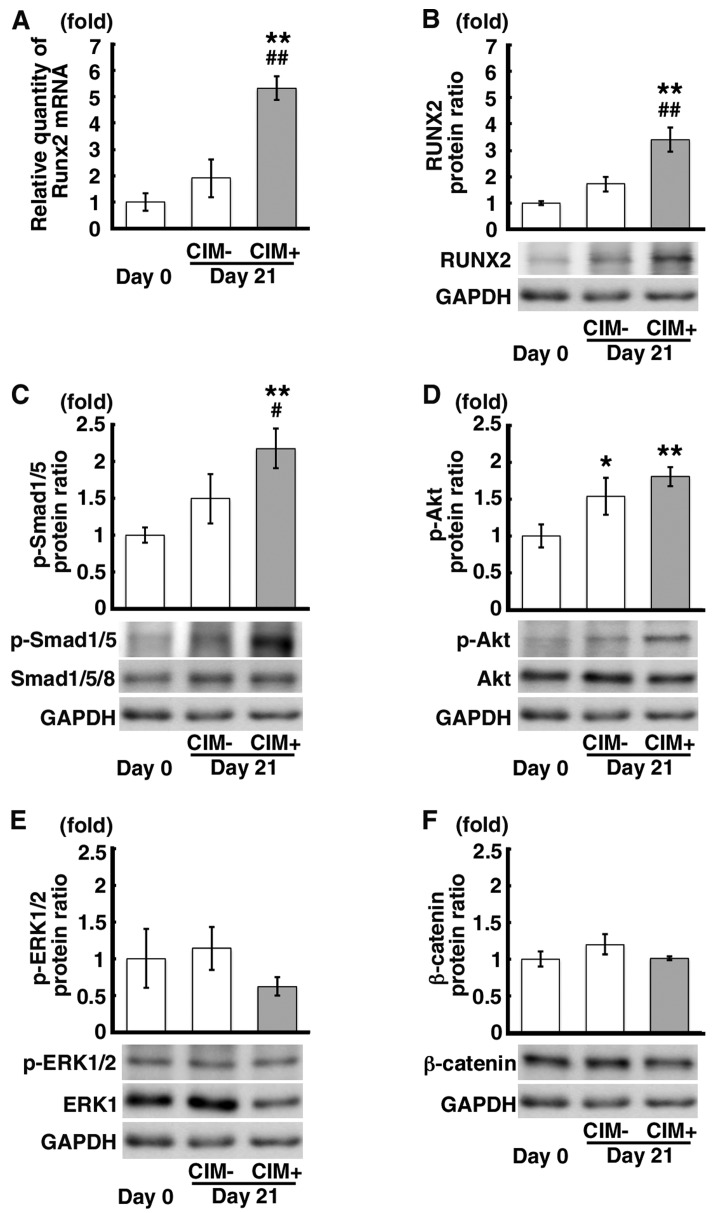
The results of the analysis of signaling pathways upstream of runt-related transcription factor 2 (Runx2) expression in the mDE6 cells. (A) The mRNA and (B) protein expression levels of Runx2 were increased in the mDE6 cells cultured in calcified induction medium (CIM) for 21 days. (C–F) The expression level of each phosphorylated/non-phosphorylated protein is shown. The expression levels of (C) p-Smad1/5 and (D) p-Akt were significantly increased in the mDE6 cells treated with CIM for 21 days compared with those in the control cells not treated with CIM. There were no significant differences observed in the (E) p-ERK1/2 and (F) β-catenin protein expression levels between the treated mDE6 cells and the controls. The data are the means ± SD from triplicate samples. ^*^P<0.05 and ^**^P<0.01 vs. day 0; and ^#^P<0.05 and ^##^P<0.01 vs. CIM- by a one-way ANOVA with the Tukey-Kramer comparison test.

In order to confirm which signaling pathway(s) is/are involved in calcification as the upstream mediator of Runx2 expression, we examined the mRNA and protein expression levels of Runx2 and the protein expression of p-Smad1/5, p-Akt, p-ERK1/2 and β-catenin in the CIM+ cells in comparison to that observed in the CIM-cells and the cells on day 0. The mRNA and protein expression levels of Runx2 were significantly increased in the CIM+ cells in comparison to those observed in the CIM- and the cells on day 0 (Fig. 2A and B). The p-Smad1/5 protein level was also increased in the CIM+ cells in comparison to that observed in the CIM- cells and the cells on day 0 (Fig. 2C). Although the p-Akt protein expression level was increased in the CIM+ cells compared to that observed in the cells on day 0 (Fig. 2D), there were no significant differences in p-Akt protein expression between the CIM+ cells and CIM- cells (Fig. 2D). Although there was a decrease in the p-ERK1/2 protein expression in the CIM+ cells and no marked changes were observed in β-catenin protein expression, there were no significant differences observed in these levels between the CIM+ cells and the controls (Fig. 2E and F). These findings suggest that the Smad signaling pathway is associated with RUNX2 expression in the mDE6 cells.

### Inhibition of the Smad and PI3K-Akt signaling pathways upstream of RUNX2 expression in mDE6 cells

**Figure 3:**
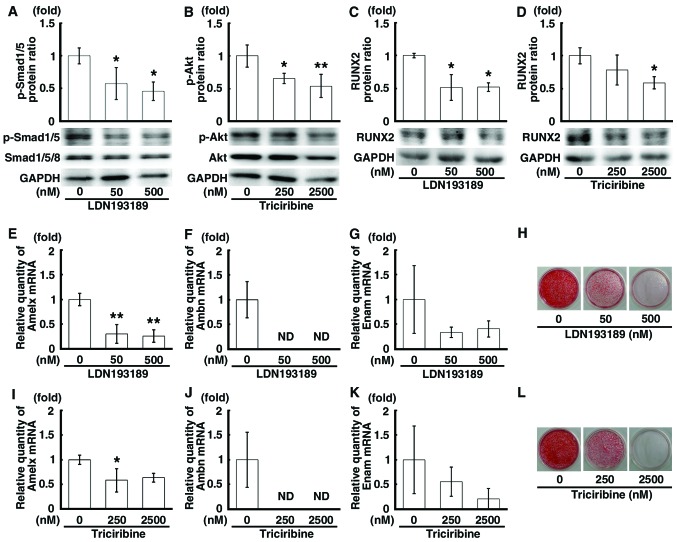
The decrease in runt-related transcription factor 2 (RUNX2) expression and calcification induced by inhibitors of the Smad (LDN193189) and PI3K-Akt (triciribine) signaling pathways in the mDE6 cells. (A and B) The expression levels of (A) p-Smad1/5 and (B) p-Akt were decreased in the mDE6 cells treated with LDN193189 and triciribine, respectively, compared with the controls. (C and D) The protein expression of Runx2 was inhibited by treatment of the mDE6 cells with (C) LDN193189 and (D) triciribine. (E–G) The mRNA expression levels of (E) amelogenin, X-linked (Amelx), (F) ameloblastin (Ambn) and (G) enamelin (Enam) were decreased by treatment with LDN193189. (H) The mDE6 cells were incubated with calcified induction medium (CIM) containing LDN193189 for 10 days. Calcification was suppressed by treatment with LDN193189 for 10 days. (I–K) The mRNA expression levels of (I) Amelx, (J) Ambn and (K) Enam were decreased by treatment with triciribine. (L) The cells were incubated with CIM containing triciribine for 10 days. Calcification was suppressed by treatment with triciribine for 10 days. The data are the means ± SD from triplicate samples. ^*^P<0.05 and ^**^P<0.01 vs. control by a one-way ANOVA with the Tukey-Kramer comparison test. ND, not detectable.

By using 2 different inhibitors of the Smad (LDN193189) and PI3K-Akt (triciribine) signaling pathways, we examined the association between the Smad and PI3K-Akt signaling pathways and the expression of RUNX2 in the mDE6 cells. These agents prevent the phosphorylation of Smad1/5/8 and Akt, respectively. Following treatment with the inhibitors for 48 h, the protein expression levels of p-Smad1/5 and p-Akt were significantly decreased in a concentration-dependent manner by LDN193189 or triciribine (Fig. 3A and B). A decrease in the protein expression level of RUNX2 was also observed in the cells treated with LDN193189 or triciribine (Fig. 3C and D). The mRNA expression levels of Amelx, Ambn and Enam were markedly decreased in the cells treated with these inhibitors (Fig. 3E–G and I–K). Of note, the mRNA expression of Ambn was ‘not detectable’ (Fig. 3F and J). Moreover, when the mDE6 cells were cultured in CIM with LDN193189 or triciribine for 10 days, the formation of calcified material was attenuated in a concentration-dependent manner in the treated cells (Fig. 3H and L). Thus, the Smad and PI3K-Akt pathways are necessary for the expression of odontogenesis-related genes, including Runx2, as well as for the calcification of the mDE6 cells.

### Effects of the siRNA-mediated Tb4 knockdown on RUNX2 expression and calcification of the mDE6 cells

**Figure 4:**
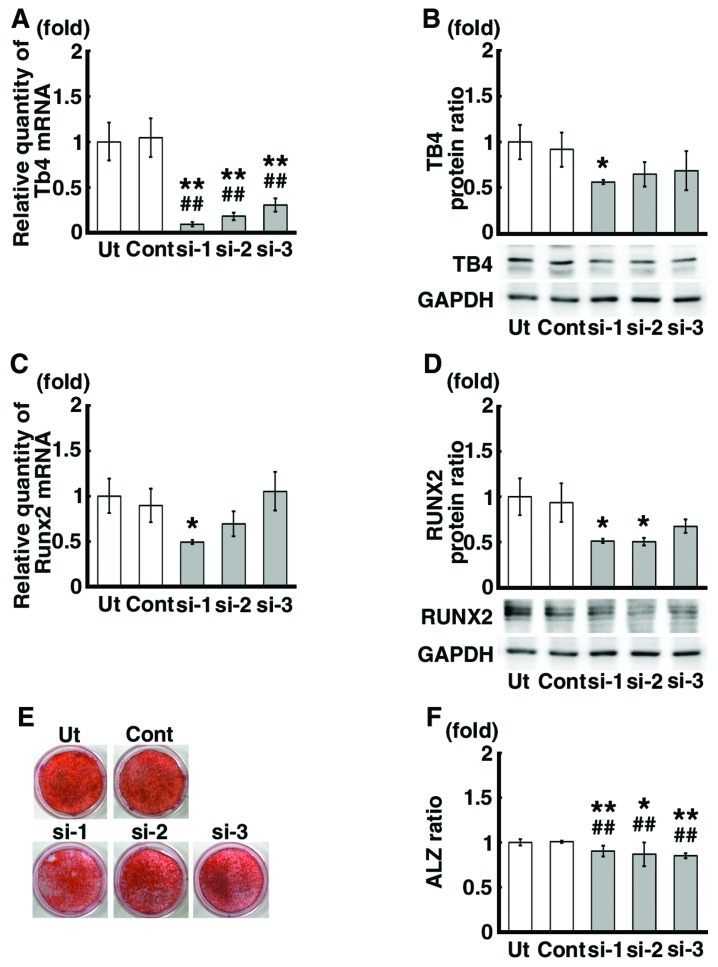
Effects of siRNAs against thymosin beta 4 (Tb4) on the runt-related transcription factor 2 (RUNX2) expression and calcification in the mDE6 cells. (A) The Tb4 mRNA expression was significantly decreased in the mDE6 cells treated with siRNA against Tb4 in comparison to that observed in the untreated mDE6 cells (Ut), or mDE6 cells treated with a universal negative control siRNA (Cont). (B) Similarly, the protein expression level of TB4 was decreased following Tb4-siRNA treatment. (C) The Runx2 mRNA expression level was decreased in the samples treated with siRNA-1 and -2, not -3. (D) The RUNX2 protein expression level was decreased in the samples treated with siRNA-1, -2 and -3. (E) The mDE6 cells cultured in calcified induction medium (CIM) were treated with siRNAs against Tb4 for 10 days. Calcification was decreased in the mDE6 cells treated with these siRNAs in comparison to that observed in the Ut and Cont cells. (F) The ratio of the Alizarin red S (ALZ)-positive area/dish bottom area was significantly reduced in the cells treated with siRNAs against Tb4 in comparison to that observed in the Ut and Cont cells. The values represent the means ± SD of 3 independent experiments. ^*^P<0.05 and ^**^P<0.01 vs. Ut; and ^##^P<0.01 vs. Cont by a one-way ANOVA with the Tukey-Kramer comparison test.

Transfection of the cells with 3 different siRNAs against Tb4 (siRNA-1, -2 and -3) significantly decreased the mRNA expression levels of Tb4 by approximately 70–90% compared to those in the untreated control mDE6 cells (Ut) and the mDE6 cells treated with a universal negative control siRNA (Cont) (Fig. 4A). The protein expression level of TB4 was also decreased by approximately 40–50% (Fig. 4B). The mRNA expression level of Runx2, which has been suggested to be one of the downstream genes of Tb4 (17,19), tended to decrease in the mDE6 cells treated with siRNA-1, and -2, not -3 (Fig. 4C). A significant decrease in the Runx2 mRNA expression level was observed in the siRNA-1 treated cells (Fig. 4C). The protein expression level of RUNX2 was also significantly decreased in the mDE6 cells treated with siRNA-1 and -2 (Fig. 4D), although no significant differences in the RUNX2 protein expression level were noted between the siRNA-3 treated cells and the controls (Fig. 4D). When the mDE6 cells cultured in CIM were treated with siRNA-1, -2 or -3 for 10 days, the formation of ALZ-positive calcification was slightly decreased (Fig. 4E). The ratio of the ALZ-positive area to the total area (ALZ ratio) was significantly reduced in the siRNA-treated cells (Fig. 4F).

### Effects of siRNA against Tb4 on the signaling pathways upstream of Runx2 expression in mDE6 cells

**Figure 5:**
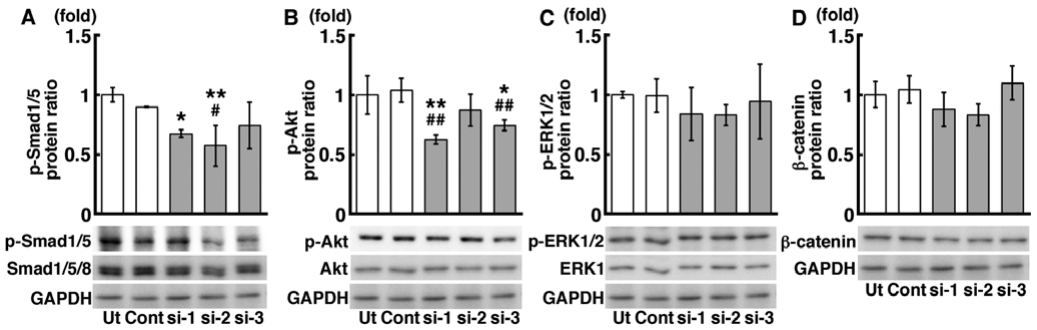
The results of the analysis of the signaling pathways upstream of runt-related transcription factor 2 (Runx2) expression in the mDE6 cells treated with siRNAs against thymosin beta 4 (Tb4). (A-D) The expression levels of (A) p-Smad1/5 and (B) p-Akt were decreased in the mDE6 cells treated with Tb4-siRNAs compared with those in control cells. Although the expression levels of (C) p-ERK1/2 and (D) β-catenin showed a tendency to be decreased in the mDE6 cells treated with Tb4-siRNAs, there were no significant differences observed. The data are the means ± SD from triplicate samples. ^*^P<0.05 and ^**^P<0.01 vs. Ut; and ^#^P<0.05 and ^##^P<0.01 vs. Cont by a one-way ANOVA with the Tukey-Kramer comparison test.

We analyzed the effects of siRNA against Tb4 on the signaling pathways upstream of Runx2 expression in the mDE6 cells. The protein expression levels of p-Smad1/5 and p-Akt were significantly decreased in the Tb4-siRNA treated cells (Fig. 5A and B), although the degree of decrease varied depending on the siRNA used (siRNA-1, -2 and -3). No significant differences were observed in the expression levels of p-ERK1/2 and β-catenin between the Tb4-siRNA treated cells and the controls (Fig. 5C and D).

**Figure 6:**
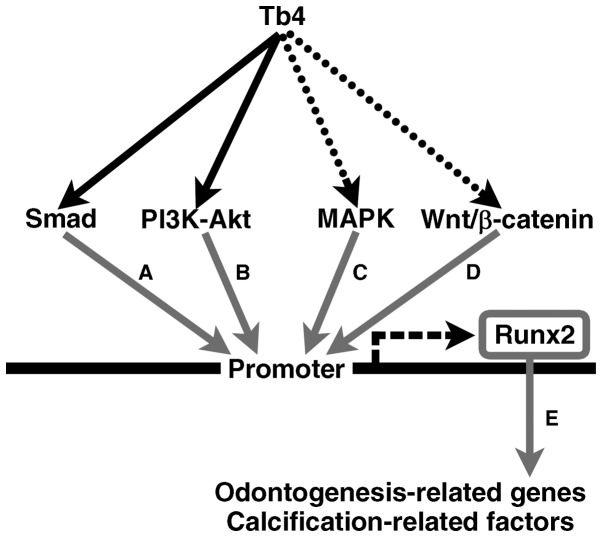
Schematic illustration of the putative signaling pathways from thymosin beta 4 (Tb4) to runt-related transcription factor 2 (RUNX2) in the mDE6 cells. Several signaling pathways through Smad, PI3K-Akt, MAPK and/or Wnt/β-catenin have been reported to be upstream of RUNX2 expression. The results of this study suggest that Smad and PI3K-Akt may participate in Tb4-RUNX2 signaling pathway(s) in the mDE6 cells. Tb4 may increase RUNX2 expression to induce the expression of odontogenesis-related genes in the mDE6 cells. The black arrows indicate putative Tb4-RUNX2 signaling pathways revealed in this study, while the dotted arrows indicate possible Tb4-RUNX2 signaling pathways that were not supported in this study. The gray arrows indicate signaling pathways reported in previous studies [(A) (34,42); (B) (34,43); (C) (34,44); (D) 34,45; (E) (9,17,19,29–31)]. RUNX2 can upregulate the expression of downstream biological effectors, including odontogenesis-related genes and calcification-related factors.

The data from our study indicate the putative signaling pathways from Tb4 to Runx2 expression in the mDE6 cells. The Smad and PI3K-Akt signaling pathways also appeared to play a role in the Tb4-RUNX2 pathway in mDE6 cells (Fig. 6). However, little is known as to whether upregulated Tb4 can induce the expression of odontogenesis-related genes, such as Amelx, Ambn and Enam, without the expression of Runx2, and of the association between Tb4 and other signaling pathways upstream of Runx2 expression.

## Discussion

The tooth comprises hard matrices consisting of enamel, dentin and cementum as the outer parts, and the dental pulp soft tissue as the central part of the tooth. As a result of sequential and reciprocal epithelial-mesenchymal interactions, the dental epithelium differentiates into ameloblasts, which secrete enamel matrix. In our previous studies, we suggested that Tb4 plays an important role in the development of the tooth germ through Runx2 expression. Tb4 appeared to be associated with the differentiation of the dental epithelium (8,17,19). In the present study, we suggest that Tb4 is associated with RUNX2 expression through the Smad and PI3K-Akt signaling pathways, and with calcification through RUNX2 expression in mDE6 cells.

The present study, as well as our previous studies suggested that Tb4 affects Runx2 expression in dental epithelial cells and in the developing tooth germ (17,19). Although accumulating evidence suggests that Runx2 is a key gene involved in tooth development, and that it can lead to the expression of odontogenesis-related genes, such as fibroblast growth factor 3 (Fgf3), Sonic hedgehog (Shh), Amelx, Ambn, Enam, dentin matrix acidic phosphoprotein (Dmp) and dentin sialophospho-protein (Dspp) (9,17,19,29–31), little has been reported on the mechanism(s) underlying the signaling pathways from Tb4 to Runx2 in dental epithelial cells. This prompted us to investigate this signaling.

Tb4 is an actin-binding peptide known to regulate the polymerization and depolymerization of actin (23), but does not contain the DNA-binding, signal-sensing, or transactivation domains found in a prototypical transcription factor (40). However, Tb4 appears to upregulate a number of biological effectors, such as vascular endothelial growth factor (VEGF), laminin-5 (23) and transforming growth factor (TGF)β. Tb4 has been reported to translocate from the cytoplasm into the nucleus (41). Therefore, a transcription factor mediator activity for Tb4 has been suggested. Thus, in this study, we examined which signaling pathway(s) associated with RUNX2 expression is/are affected by Tb4 expression in dental epithelial cells.

In this study, we investigated the association between Tb4 and Smad, Akt, ERK and/or β-catenin in dental epithelial cells, as the signal pathways of these factors are associated with Runx2 expression during bone differentiation (42-45). In addition, the Smad, PI3K-Akt, MAPK and Wnt/β-catenin signaling pathways are associated with tooth development (21,36,46,47), but it is unclear as to whether these pathways are mediated by Tb4 and affect Runx2 expression. mDE6, a mouse dental epithelial cell line, was used in this study, as we confirmed that mDE6 cells expressed some odontogenesis-related genes and formed calcified material with enamel formation following culture in CIM (Fig. 1). Following the induction of calcification and treatment with inhibitors of the p-Smad1/5/8 and p-Akt pathways, our results revealed that the p-Smad1/5 and p-Akt signaling pathways were required for the induction of Runx2 expression and the expression of Amelx, Ambn and Enam. When either of these pathways was inhibited, the calcification of the mDE6 cells was markedly suppressed (Fig. 3). These results are in accordance with those of the study by Hu *et al* (42), which indicated that the expression of Runx2 was significantly reduced by LDN193189 (final concentration, 500 nM) in bone marrow stromal cells. The activity of Smad1/5/8 is regulated by bone morphogenic protein (BMP)-2 and -4, and affects tooth development (48). Takayama *et al* (49) also reported that enamel matrix derivative stimulates Runx2 expression through the activation of Smad1 in mouse myoblast cells. BMP-2 has previously been shown to induce the expression of Amelx and Ambn in ameloblast-like cells (50). The Smad signaling pathway contributes to Runx2 and odontogenesis-related gene expression in tooth development.

On the other hand, a study on the PI3K-Akt signaling pathway reported that this pathway plays a role in the differentiation and proliferation of odontogenic tumors (47). Although little is known about the activity of the PI3K-Akt signaling pathway during tooth germ differentiation, the present study revealed that the PI3K-Akt signaling pathway plays an important role in the induction of the expression of Runx2, Amelx, Ambn and Enam and in the calcification of mDE6 cells. No apparent differences were observed following the inhibition of the ERK and β-catenin pathways in our study (data not shown). These results suggest that not only the Smad signaling pathway, but also the PI3K-Akt signaling pathway, are important for the induction of Runx2, Amelx, Ambn and Enam expression that occurs during the calcification of mDE6 cells.

Moreover, the knockdown of Tb4 expression using siRNA significantly reduced the Runx2 expression level and the degree of calcification of the mDE6 cells (Fig. 4). The protein expression of p-Smad1/5 and p-Akt was decreased in the mDE6 cells in which Tb4 was knocked down (Fig. 5). Previous studies have reported that Akt is phosphorylated by Tb4 (51), and that BMP-2 and -4 are also activated by Tb4 to induce Smad1/5/8 signaling (21). This suggests that Tb4 participates in the calcium deposition through Runx2 expression during tooth development, which is compatible with the findings of our previous studies (17,19). However, despite the marked decrease in Tb4 expression induced by siRNA against Tb4 in the mDE6 cells in the present study, the degree of restraint of Runx2 expression and calcification was minor (although significant) compared to that observed in our previous study (19). This may have been caused by the off-target effects of the methods used, the characteristics of the specific cell line and so on. The mDE6 cells used in this study were established from dental epithelial cells of mouse tooth germ, and have some of the characteristics of ameloblasts. In our previous study, non-odontogenic keratinocytes were transfected with a Tb4 expression vector to enforce the expression of Tb4, which led to the expression of some odontogenesis-related genes (19). Factors other than Tb4 may also be associated with the Runx2 expression in the mDE6 cells. During the induction of calcification, the CIM may directly upregulate Runx2 expression and calcification and/or may indirectly upregulate its expression through interactions with other factors. The reason for these differences is currently unknown. The interaction of other, currently unknown, factors in mDE6 cells may affect Runx2 expression and calcification. However, the present study suggests that Tb4 has the ability to upregulate the expression of downstream biological effectors, including Amelx, Ambn and Enam in the mDE6 cells, as well as in the tooth germ (17) and non-odontogenic keratinocytes (19).

In conclusion, the present study suggests that Smad and PI3K-Akt signaling pathways play important roles in expression of Runx2 induced by Tb4, which is an important factor involved in the development of the tooth germ, in mDE6 cells (a mouse dental epithelial cell line). These signaling pathways may be tightly associated with the expression of odontogenesis-related genes, such as Amelx, Ambn and Enam, and the formation of calcified material through Runx2 expression. It is unknown whether the Smad pathway or PI3K-Akt signaling pathway are the main pathways involved. Further studies are required to identify the detailed signaling cascades associated with tooth development. Although little is known of the Tb4-mediated upregulation of odontogenesis-related gene expression in the absence of Runx2 expression, and of the association between Tb4 and the other signaling pathways upstream of Runx2 expression, this study provides new information concerning the putative signaling pathways from Tb4 to Runx2 expression in mDE6 cells, which may be helpful in understanding the optimized regulation and improving the success rate of tooth development and regeneration.

## Figures and Tables

**Table I:** Primers used in RT-qPCR.

Gene name	Accession no.	Primer sequences
Gapdh	NM_001289726.1	F: 5′-TGT GTC CGT CGT GGA TCT GA-3′
	NM_008084.3	R: 5′-TTG CTG TTG AAG TCG CAG GAG-3′
Tb4	NM_021278.2	F: 5′-CTG ACA AAC CCG ATA TGG CTG A-3′
		R: 5′-ACG ATT CGC CAG CTT GCT TC-3′
Runx2	NM_001146038.2	F: 5′-GGT TAA TCT CTG CAG GTC ACT ACC A-3′
	NM_001271627.1	R: 5′-ACG GTG TCA CTG CGC TGA A-3′
	NM_009820.5	
Amelx	NM_009666.4	F: 5′-AGC ATC CCT GAG CTT CAG ACA GA-3′
		R: 5′-AAC CAG GGC TTC CAG GAT GAG-3′
Ambn	NM_009664.1	F: 5'- CCT GGG AGC ACA GTG AAT GTC-3'
		R: 5′-TCA AAC TAG CCA TGC CAG GAG-3′
Enam	NM_017468.3	F: 5′-CCG AAT GCC TGG ATT TAG CAG TA-3'
		R: 5'-GGG TTG CTG CCA TCC ATT G-3′

Gene name, accession number and sequence were provided by the RefSeq database of the National Center for Biotechnology Information. Gapdh, glyceraldehyde-3-phosphate dehydrogenase; Tb4, thymosin beta 4; Runx2, runt-related transcription factor 2; Amelx, amelogenin, X-linked; Ambn, ameloblastin; Enam, enamelin; F, forward; R, reverse.

**Table II:** Antibody types and source.

Target protein	Provider ID	Antibody	Dilution
GAPDH	SC20357	Goat Polyclonal IgG	1:1000
TB4	SC67114	Rabbit Polyclonal IgG	1:1000
RUNX2	AB76956	Mouse Monoclonal IgG2a	1:1000
Smad1/5/8	SC6031	Rabbit Polyclonal IgG	1:1000
p-Smad1/5	CST9516	Rabbit Monoclonal IgG	1:2000
ERK1	BD610030	Mouse Monoclonal IgG1	1:8000
p-ERK1/2	BD612358	Mouse Monoclonal IgG1	1:4000
Akt	CST4691	Rabbit Monoclonal IgG	1:2000
p-Akt	CST4060	Rabbit Monoclonal IgG	1:2000
β-catenin	CST9582	Rabbit Monoclonal IgG	1:4000

AB, Abcam (Cambridge, UK); BD, Becton, Dickinson and Company (Franklin Lakes, NJ, USA); CST, Cell Signaling Technology (Danvers, MA, USA); SC, Santa Cruz Biotechnology, Inc. (Dallas, TX, USA); GAPDH, glyceraldehyde-3-phosphate dehydrogenase; TB4, thymosin beta 4; RUNX2, runt-related transcription factor 2.
